# A Materialist Converted

**Published:** 1889-03

**Authors:** Chas. Duckworth


### A MATERIALIST CONVERTED.

I wish to give to the world my experience, as a hunter of information on spiritualism. While talking with a friend on the street in Chicago, he related to me an experience he had with one Eugene Stevenson, of 922 Fifth ave., Minneapolis, a medium for answering sealed letters. My friend was so profuse in his praises, I concluded to show him how completely he had been duped.

You must understand I have been a rabid Materialist, for twenty-five years, I took no stock in Spiritualism, having written several sealed letters to mediums, with very unsatisfactory results. Hence, it was with a self-satisfied feeling of success in my undertaking to show my friend the duplicity of the medium he so highly extolled, that I prepared a letter to read to him. After writing twelve questions that none but the deceased relative to whom they were addressed could answer, I cemented the edges together with white furniture glue ; then after stitching it around the edge inside the glue, I placed it in an opaque envelope, which I also glued. I then had the seams well covered with sealing wax, and on the wax made eight impressions of my seal ring, and the end of a trunk key. All this I enclosed in a large envelope, with a substituted name and address, and mailed it the first day of October.

On the 26th of that month I received a reply, also my letter, which I did not open until I had the audience of my friend. Upon opening it in the presence of my friend, what was my surprise to find the questions not only correctly, but minutely answered For example, question No. 3, was : "Where and of what disease did you die?" Answer : "I died in Augusta, Maine, Dec. 27, 1859. I died of no disease, but from wounds made by the accidental discharge of a shot-gun. I died the 4th day after the wounds were received, at the house of my Uncle Andrew." This was correct in every particular. The other questions were as satisfactorily answered, and as there was nothing else for me to do, I dropped my Materialism and became a firm believer in Mr. Stevenson's spiritualistic phenomena. My business calls me to this part of the country about four months out of the year, and since my arrival here, I have had several independent slate-writing sittings with Mr. Stevenson, and the genuineness of his accomplishments is beyond question, as the writing came between slates held by myself. Respectfully yours reformed,

CHAS. DUCKWORTH (in *New Thoughts*).

